# What is public health? public goods, publicized goods, and the conversion problem

**DOI:** 10.1007/s11127-021-00908-8

**Published:** 2021-06-03

**Authors:** Jonathan Anomaly

**Affiliations:** Philosophy, Politics, and Economics Program, University of Pennsylvania, Philadelphia, USA

**Keywords:** Public health, Public goods, Public policy, H10, H40, H41, H42, H51, I18

## Abstract

Public health programs began as an attempt to fight infectious diseases that are difficult to address without collective action. But the concept and practice of public health has ballooned to encompass an expanding list of controversial public policy goals ranging from reducing obesity to raising self-esteem. As the list of controversial goals expands, support for “public health” measures contracts. I’ll briefly defend the view that we should define public health as the provision of health-related public goods. I’ll then show that being a health-related public good is not a sufficient condition for counting as a public health goal, since virtually any private good can be converted into a public good by government fiat. This is the conversion problem, which challenges the way we ordinarily think about public goods and public health.

## Introduction

According to the traditional view, public health should be thought of as the provision of health-related public goods (Epstein, 2004).^1^ On that view, a program promotes *public* health rather than *private* health when it supplies health-related goods that^2^ (a) are available to everyone in a population, (b) informed citizens consider desirable, and (c) require collective action to produce (Anomaly, 2011). According to critics of the traditional view, we should either expand public health to cover all health-related market failures (Horne, 2019), or we should reconceive public goods as morally desirable outcomes that governments can achieve by exercising their coercive power (Dees, 2018).

In this essay, I will briefly defend the public goods view of public health. But I’ll mainly focus on what we might call “the conversion problem”. The conversion problem occurs when a private good is converted into a public good, usually because a government policy spreads the costs and benefits of risky choices onto everyone in a population.^3^ For example, consider what happens when governments require citizens to purchase home insurance, but then forbids insurance companies from charging higher prices when people build in places that are prone to earthquakes or floods. Under that mandate, refraining from building homes in risky areas is a public good: individual restraint provides nonrival, nonexcludable value to everyone in the insurance pool.^4^ In fact, once the insurance mandates are in place, policymakers could argue credibly on public goods grounds that they should either prevent people from building in certain areas, or pay people to avoid those areas.

A more salient case that I’ll discuss below involves public health professionals designating obesity and smoking as public health problems. Public health officials often say smoking and obesity impose costs on a national health service or an insurance pool. But they usually fail to note that rules mandating sharing the costs and benefits of one another’s health decisions are themselves a function of public policies that transform what would otherwise be private habits into public concerns. As those examples show, many modern debates in public health concern *publicized* goods—goods that are made public through the incentives created by government mandates. Since publicized goods are ubiquitous, even on the public goods conception of public health, there is no timelessly true answer to what constitutes a public health program. What counts as a public health program will depend on the normative judgments of the citizens who comprise a particular polity in conjunction with the incentives their political institutions create.

## Defending the public goods account of public health

One challenge to the public goods account of public health is that it is too narrow. For example, everyone agrees that vaccination campaigns and chlorinated water supply health-related public goods that reduce the prevalence of infectious diseases. But according to Richard Dees (2018), the public goods account wouldn’t justify governments removing toxins from the water supply (e.g., mercury and arsenic, which can adversely affect the neurological development of children). That is because, “unlike those without pathogen-free water, those without toxin-free water do not threaten those with it” (ibid., p. 22). Since providing clean drinking water to a population strikes most people as an obvious case of a public health program, Dees thinks it would be odd to exclude it as a case of public health.

It is true that supplying toxin-free water to citizens is different than removing infectious agents from a water supply. Toxins like mercury or arsenic do not use people as vectors to spread disease in the way viruses, bacteria, and other parasites do. Nevertheless, supplying information about the dangers of certain chemicals, or making water without those chemicals available to whole populations, can count as health-related public goods—and thus as a public health program—even when the purpose is not to prevent the spread of infectious diseases. When the state supplies information about the health effects of consuming dangerous chemicals, or removes pollution from a public water supply, the typical justification is that they are using coercion to achieve a widely desired outcome in a more efficient way than each person acting alone could achieve that outcome. This is the essence of the traditional rationale for governments to supply public goods (Pigou, 1920).

Of course, private water supplies exist, including backyard wells, and in the age of the internet, many people can learn what kinds of chemicals they should avoid adding to drinking water if they want to drink healthy water. But good reasons can also be found for the state to supply health information or to use its power more directly to create a system of canals and chlorination to make clean water available to all. Those reasons do not stem from controversial moral goals, but from the fact that if we understood the situation fully we would agree that the central supply of clean water, which often requires coercion to produce efficiently, is better for nearly everyone in a population than relying on individual effort and ingenuity. In other words, the supply of clean drinking water is a non-rival, non-excludable good that benefits nearly everyone in a population.

To be sure, some governments are corrupt, and some societies have low social trust (Vallier, 2021). In such circumstances, it may be the case that private efforts to provide clean drinking water will be more effective than government supply.^5^ But the point is that governments acting to supply clean drinking water can easily be justified on public goods grounds within a classical welfare economics framework—namely, sometimes the state can help people achieve collective goals more effectively than they would if each were left to their own devices.

However, according to Richard Dees (2018, p. 23), even if we accept something like a public goods account of public health, public health should be concerned with the provision of *normative* public goods:

[A] normative public good must meet four requirements. (i) It must be a good. (ii) The good should be readily accessible to everyone in a way that individuals need not worry about using it up… (iii) It must benefit society… (iv) The good must be sufficiently important to justify the collective effort. What will count as ‘important’ and how many people constitute ‘large number’ are left open to debate”.

As Dees knows, the word “important” embodies a value judgment. One of the main reasons to confine public health to public goods for which widespread demand exists^6^ is that it avoids the need to make controversial value judgments. The view Dees endorses is quite consistent with the direction in which the public health profession has been moving: toward regulating smoking, obesity, and alcoholism, reducing prejudice and economic inequality, and a number of other progressive causes. In all such cases, some people think that government should take an active role in altering what kinds of food people eat, which drugs they use, whether they harbor prejudice against other groups, or earn more money than average. Since our beliefs about how other people should live are based on moral commitments, it makes sense that some citizens would try to implement these commitments using a public health justification.

However, one reason to reject promoting controversial moral goals under the guise of “public health” is that it tends to erode the perceived authority of public health professionals. For example, when the novel coronavirus outbreak of 2020 spread from its source in China, citizens around the world were ordered to stay home. But when people filled the streets in American cities to protest perceived racial injustice in the summer of 2020,^7^ many public health officials and university faculty declared their support for what became violent riots, with some saying that the unrest was unlikely to spread the virus (Richardson, 2020). It seemed like an obvious case in which political goals clouded the scientific statements of public health officials, and many people became concerned that public health officials were overstepping their authority and promoting controversial social goals. It is plausible that the more we conflate public health with sectarian political goals, the less will be the support for *all* public health programs, which may lead to distrust in officials tasked with promoting important goals that benefit everyone, such as vaccination campaigns.^8^

Another challenge to the public goods conception of public health is that it focuses on only one of many sources of market failure (Horne, 2019). While providing some public goods can increase social welfare, the same can be said for solving problems associated with externalities, information asymmetries, adverse selection, and other cases in which (at least in principle) the free exchange of goods tends to produce less social welfare than would be achieved in the presence of coercive government mandates.

While many market failures are associated with health care, not all of them produce population-level effects. For example, smoking increases various health risks, and people who smoke in outdoor spaces produce obnoxious smells, which are negative externalities. But they do not produce enough pollution to endanger significant numbers of people in the population (if someone is smoking in an uncrowded outdoor space, it is easy enough to walk away, just as it is easy enough to walk away from someone wearing strong perfume). If, by contrast, people are permitted to smoke indoors, including in grocery stores and office buildings, adverse population-level effects on people’s health may be created. Banning smoking in densely packed places that are hard to avoid is consistent with the public goods account because it produces health benefits that affect nearly everyone in a population. In that case, however, the externality problem can be re-described as a public goods problem because the relevant externalities are non-excludable, non-rival and borne by large numbers of people.

Similar considerations apply to information asymmetries. If health care providers have information that patients cannot access, and it enables them to exploit patients by fooling them into undergoing procedures they wouldn’t consent to if they understood the risk, then remedying such information asymmetries plausibly improves patient health outcomes. In any particular case, treatment may be a private good for a particular patient. But notice that, as in the externality case, if a policy makes information available to all patients, and that information enables them to make better choices with respect to their own health, we can redescribe fixing the information asymmetry as a health-related public good form which nearly everyone in a population benefits.

One *conceptual* virtue of the public goods account of public health is that it is clear and simple. A *moral* virtue of the public goods account of public health is that it avoids controversial normative judgments because it only justifies government interventions that attempt to improve population health in ways to which nearly everyone would consent. Sometimes correcting externalities and information asymmetries achieves that goal. But policies that fix market failures seem relevant only to *public* rather than *private* health when they affect entire populations. And in those cases, they can be considered public goods. This is not an argument against state intervention to solve market failures in health care markets. It is only an argument that not all such interventions should be thought of as cases of public health.

## How private goods become public goods, and why it matters for public health

Assume that the public goods account of public health is plausible. As spartan as the account is, it is still far too inclusive. That is because what counts as a public good is partly a function of the cost and feasibility of excluding non-users from enjoying its benefits, and partly a function of whether the state has converted what would otherwise be a private good into a public good.

Consider an unusual example that has gained traction among economists: children. In a provocative article, Nobel laureate Gary Becker (1960) describes children as private consumption goods.^9^ Economist Nancy Folbre (1994) argues that children are public goods to the extent that they eventually work and pay taxes that finance social services that are available for all citizens to consume. Garett Jones (2015) takes Folbre’s argument a step further by highlighting the ways in which having smart children in a population, who eventually become smart adults, contributes to economic growth. Everyone benefits when social trust is high, scientific innovation occurs, new vaccines are invented, reliable energy becomes cheaper, and so on. And if Jones is right, those benefits are strongly correlated with the average IQ of a population. In that sense, we can think of children who become productive workers and taxpayers as public goods (Folbre’s claim), and we can think of the social benefits of children as partly a function of their intelligence (Jones’s claim).

Of course, the extent to which children are public goods also depends on general political institutions (like democracy, wherein each person’s vote has a small impact on other people) and specific public policies (like social welfare programs, to which some people contribute more than others). Put differently, when economists claim that children are public goods, they mostly mean *publicized* goods, since different policies would alter the extent to which children produce rival or excludable value. However, within almost any functional human group—whether a small tribe or a large nation—children will be public goods to some extent regardless of policies, since the size and composition of any population of people will affect the genetic trajectory and social welfare of the population.

Adam Smith observed that wealth is largely a function of trade, which is limited not only by natural barriers like mountains, and political restrictions like tariffs, but also by the quantity and quality of people in a population. Other things equal, Smith (1776, chs 2 and 5) thinks, having a larger network of people to trade with and a more educated population translates into the discovery of more ideas, and thus more social welfare.

Charles Darwin (1874, p. 140) agrees: “It is most difficult to say why one civilised nation rises, becomes more powerful, and spreads more widely, than another; or why the same nation progresses more at one time than at another. We can only say that it depends on an increase in the actual number of the population, on the number of men endowed with high intellectual and moral faculties, as well as on their standard of excellence”. In that way, a larger population, and one with greater productive potential, is a public good that underlies almost all other public goods that governments might provide, since it creates the conditions for wealth to emerge and for government to work.

Apart from the benefits of increasing human capital, people in all societies bear some of the costs of one another’s reproductive choices. As Allen Buchanan and his co-authors (2000, p. 210) put it, “significant portions of the costs of having children are externalized in virtually all societies—that is, borne by others besides the parents (or children). The more this happens, the greater a claim these others might make to have some say in, or control of, the costs imposed on them”. The same view was expressed by Supreme Court Justice Oliver Wendell Holmes, Jr., in *Buck v Bell*, when the court relied on a public health rationale to justify forced sterilizations of those who impose social costs on others through careless reproductive choices: “The principle that sustains compulsory vaccination is broad enough to cover cutting the Fallopian tubes”^10^. The more we share the costs and benefits of productive activity, the easier it is to make a public goods argument—and by extension, a *public health* argument—for the state to regulate what would otherwise be private decisions, including our reproductive choices, which generate some of the most extensive externalities of any choices that we make.

How much we feel the consequences of one another’s reproductive choices is partly a function of government programs that redistribute work in the form of taxation and subsidies for social welfare programs. How much value Albert Einstein produces is in part a function of whether a state has a functioning educational system, whether Einstein is surrounded by other smart people to whom he can talk, and whether the society in which Einstein lives is productive enough to afford him the leisure time to contemplate special relativity. Similarly, how much of a cost a mentally or physically impaired child imposes on others is partly determined by public policies that redistribute income, risk, and effort. In ancient Spartan society, physically weak children were a net cost, so an obvious solution was infanticide. In modern British society, a physically weak but mentally acute scientist like Stephen Hawking is a net benefit, even if others are paying his medical bills through the National Health Service.^11^

I am not taking a stand on what shape any particular state should take. Instead, I want to emphasize that what is a public good and what is a private good is often a function of background conditions, including government policies. One way to turn children into public goods is to compel taxpayers to finance education, health care, and welfare programs that provide those goods without direct out-of-pocket cost to all people, regardless of their choices. Once children become public goods, and once the reproductive choices that produce children become public goods, it is much easier to justify regulations surrounding reproduction as public health interventions (Anomaly, 2014).

Similar arguments can be made for regulating the choices people make about what to eat and whether to smoke. Obesity and smoking are often said to be public health problems, and the justification that’s often given for discouraging smoking or bad eating habits is the social costs smokers and fat people are said to impose on society, given the fact that we all pay into a common insurance pool or national health service (Faden et al., 2020). Under those conditions, it is claimed, people who don’t smoke or overeat provide non-excludable benefits to others in the population. One problem with that rationale is the evidence that smokers, and potentially obese people, do not impose net social costs over their lifetimes (Barendregt et al., 1997; Finkelstein & Yang, 2011). Even if they impose more annual costs to health insurance systems, they actually may save more money than they spend since they live shorter lives, and most health care costs are incurred by elderly people in their last few years of life. No matter how the calculus comes out, the primary point is that the population-level benefits of individual healthy behavior is a *publicized* good: a good that is made public because of background regulations that socialize the consequences of what would otherwise be private choices.

As with the previous example involving reproductive choices, I do not want to defend the moral desirability—or economic efficiency—of rival health care systems. Instead, I want to emphasize that the incentives produced by health care mandates shape whether actions like smoking and eating are public goods or private goods, and thus whether regulations to change those behaviors should be considered part of public health or private health.

## “Bad” public goods

The economic term “public good” frequently has been misunderstood, and some of the confusion results from the informal ways in which economists have used it. For example, James Buchanan (1975, p. 11) says that “Politico-legal order is a public good; disorder is a public bad.” What he means, of course, is that social order is a good that positively affects everyone in a population, and disorder negatively affects everyone. Similarly, according to Albert Hirschman (1970, p, 101),

The distinguishing characteristic of [public] goods is not only that they *can* be consumed by everyone, but that there is *no escape* from consuming them unless one were to leave the community by which they are provided. Thus, he who says public goods says public evils. The latter result not only from the universally sensed inadequacy in the supply of public goods, but from the fact that what is a public good for some—say, a plentiful supply of police dogs or atomic bombs—may well be judged a public evil by others in the same community.

While it is technically more accurate to contrast public goods with private goods, it is obvious what those scholars mean by “public bad” or “public evil”: sometimes governments create outcomes that are considered bad by many people, and they cannot escape the costs imposed on them. Whether we call them “bad public goods” or “public bads” is not especially important, although it is usually worth using the economic term “good” in a neutral way, since it is generally used to refer to anything that we might consume, not something that is considered valuable by most people.

The real interest in raising the issue of nomenclature is to emphasize the conceptual point that most public goods we can think of do not benefit everyone, and even when they do benefit everyone, they do not benefit everyone in such a way that the benefits to each person of a coercively supplied public good exceed the costs to each person. For example, some public health programs encourage women of a certain age to get mammograms to screen for breast cancer. Some women are more susceptible to that disease than others, and so some are more likely to benefit than others. To the extent that the information campaigns targeting middle-aged women are financed by everyone, the benefits received are uneven, and for some women the costs of going to the doctor for a screening outweigh the benefits. Perhaps, though, the collective benefits are worth the collective costs, especially if we’re sharing resources in an insurance pool or a national health service.

One challenge associated with *publicized* goods, especially those with uneven benefits, is that they often conceal the underlying structure of a problem. Many people take the health care system in their country for granted, and then argue from where they are now that governments should pass a particular policy to improve individual or social welfare. In the United States, for example, health insurance is financed partly by employers. Employer-financed health insurance affects whether people will accept or leave a job, since health benefits are often a substantial fraction of a worker’s compensation package. The aggregate effects of the system are felt by all, if nothing else in the stickiness it creates in the labor market. But rather than fundamentally rethinking how health insurance (or health care) should be provided, most citizens take the existing system for granted and support small tweaks to it to ensure that they don’t lose their health coverage when they change jobs. Similarly, policymakers have little ability to change the system fundamentally because of pressure by lobbyists and citizens.

All people might benefit from a different bundle of health care mandates, but each might advocate smaller and less efficient changes to the existing bundle—perhaps because he is rationally ignorant about the alternatives, because he is only thinking about his own health, or because he thinks (perhaps correctly) that it is politically infeasible to alter the current health care leviathan. If so, the incentives created by current policy, and the perception of feasible alternatives, will shape the extent to which we think a *publicized* good is worth keeping in place, or trying to change.

Some people think that any move away from universal health care is morally undesirable for reasons of fairness, or politically infeasible in a government with a significant extent of democracy, which leads citizens to focus on the expressive benefits of policy while ignoring its costs (Brennan, 2017; Brennan & Lomasky, 1993). When that is true—when we think a certain amount of sharing health resources is inevitable because it enjoys popular support—it makes sense to advocate individually beneficial mandates for inclusion in a package of socially harmful policies. For example, if we think significant sharing of medical resources is either desirable or inevitable, infant health and prenatal care will be part of any public health program because the collective costs of unhealthy infants who develop disabilities in the future will be borne by all citizens. Similarly, policies that encourage physical fitness and healthy diets—whether the encouragement comes from governmental or private institutions—will be *public* rather than private health programs.

Recognizing the (near) inevitability of certain kinds of programs, such as universal health care mandates, may make citizens more likely to support government attempts to alter behavior, such as programs that aim to make people eat better, exercise more, or quit smoking. In that sense, even if we recognize that many goods are publicized goods, we might think some publicized goods are better than others, and that some public goods we wish governments wouldn’t provide might well be worth providing, given our view of what other people will support. Such judgments will reflect both our *moral* view of the proper role of the state in allocating health resources, and our *practical* view of what we think other people think is the proper role of the state,^12^ as well as our assessment of the feasibility of fundamental changes to a health care system at any given time.

Recall that the conversion problem arises when governments can transform private goods into public goods (which we’ve called *publicized* goods) by forcing us to share the costs and benefits of the consequences of our own behavior. The conversion problem is not that any new health care rule the government creates is a public good. Many health care policies, like policies in other sectors of the economy, are rivalrous goods that are influenced by the rent-seeking activities of influential political actors like pharmaceutical firms and medical associations. They benefit some people at the expense of others, even if their stated justification is to promote public health. For example, states that tax cigarettes and alcohol may very well say they’re doing so to promote public health even when their real rationale is to generate revenue they can spend on pet projects.

The conversion problem is generated by the fact that in some cases the state really does make what otherwise would be private behavior a matter of public concern. If the National Health Service is ordered to cover the cost of cosmetic surgeries that make octogenarians look like movie stars, taxpayers end up financing the pursuit of positional goods. In that fanciful scenario, *refraining* from getting face lifts and hair plugs becomes a public good whose benefits accrue to all taxpayers. There is nothing intrinsic to plastic surgery that creates a public goods problem (and therefore a public health problem). Few people think that a lack of access to free plastic surgery is a public health crisis. But once plastic surgery is subsidized by government policy, all taxpayers bear the consequences of what would otherwise be a private choice.

Recognizing the conversion problem, whereby governments transform private goods into public goods, is important for a robust moral assessment and economic analysis of the purpose of public health. Nearly everyone agrees that to count as a public health program a policy must affect entire populations, not merely a small set of individuals. And many agree that, at the very least, public health should be concerned with the provision of health-related public goods that require collective action to provide. But we should recognize that there is no single, timelessly true answer to what counts as a public health program. A lot depends on the current health system in place, the moral views of the citizens of a country, and the feasibility—both real and perceived—of rules that aim to alter behavior in ways that promote the health of a population.

## Footnotes

1 Public goods in the economic sense exhibit non-rivalry in access and non-excludability in consumption.

2 In a recent paper, Bernstein and Randall (2020) argue that the idea of a “health-related” public good is too vague to be useful. While they are right that it’s a vague term, sometimes vagueness is a virtue of a theory. The idea behind developing an account of public health is to organize thought and give a general sense of what distinguishes private health from public health, not to develop a novel or precise theory of health.

3 Vaughn Baltzly (2021) recently coined the term “publicized good” to refer to cases like this, which are called “artificial” or “secondary” public goods in Anomaly (2015).

4 By saying nonrival and nonexcludable *value* I mean the subset of public goods that produce consequences from which nearly everyone in a population benefits. Most public goods are not like that, but economists tend to focus on them since they represent a salient source of market failure. A “good” in the economic sense of the word is anything that can satisfy a desire. It is therefore important in discussing public goods—those goods that are nonrival and nonexcludable—to distinguish goods that merely affect everyone in a population from those from which most people in a population benefit.

5 Cases like this illustrate why counting as a public good is a necessary but not sufficient condition to count as public health, or to justify government regulation. The fact that an outcome is a public good does not mean that it is good for anyone in particular, let alone everyone.

6 In Anomaly (2011), I qualify that conclusion to include counterfactuals: the state has prima facie reasons to supply public goods for which demand *would be* nearly universal among reasonably informed and reflective citizens. Rational ignorance on the part of citizens often precludes them from knowing much about the effects of any particular policy on human welfare. The point of representative government is to have agents make some decisions for us even when we don’t understand the relevant problem.

7 I say “perceived” because the best evidence is that police are not especially racist, and do not disproportionately target any particular group, once background crime rates are accounted for (MacDonald, 2020). The argument that police are racist because they arrest more Blacks than Whites is as fallacious as the argument that police are sexist because they arrest more men than women. Men commit far more violent crime than women, and Blacks commit far more violent crime than Whites (or Asians), especially on a per capita basis.

8 Some argue that trust tends to fall in the particular scientists who are engaged in political activism, rather than in the overall enterprise of science (Cofnas et al., 2018).

9 Technically Becker describes children as consumption goods once income passes a certain threshold, and investment goods before this income threshold. For example, in traditional societies children would be an investment in the sense that they may supply labor in the household and act as informal social security by taking care of family members as they age.

10 Buck v Bell, 274 US 200 (1927).

11 George Bernard Shaw bit the bullet on this point. He endorsed democratic socialism, but recognized that without smart, informed, well-motivated citizens, any such project was doomed. Thus, following Plato and Aristotle, Shaw (1903, p. xxiv) thought “We must either breed political capacity or be ruined by Democracy, which was forced on us by the failure of the older alternatives. Yet if despotism failed only for want of a capable benevolent despot, what chance has Democracy, which requires a whole population of capable voters: that is, of political critics who, if they cannot govern in person for lack of spare energy or specific talent for administration, can at least recognize and appreciate capacity and benevolence in others, and so govern through capably benevolent representatives? Where are such voters to be found today? Being cowards, we defeat natural selection under cover of philanthropy: being sluggards, we neglect artificial selection under cover of delicacy and morality.”.

12 Voting and lobbying are likely to incorporate elements of a Keynesian beauty contest, according to which we’re voting not for the woman we think is most beautiful, but for the woman we think most people think is most beautiful.
